# Puzzles in modern biology. V. Why are genomes overwired?

**DOI:** 10.12688/f1000research.11911.2

**Published:** 2017-08-14

**Authors:** Steven A. Frank

**Affiliations:** 1 Department of Ecology and Evolutionary Biology, University of California, Irvine, CA, 92697-2525, USA

**Keywords:** Gene regulation, complex traits, artificial intelligence, deep learning, induction

## Abstract

Many factors affect eukaryotic gene expression. Transcription factors, histone codes, DNA folding, and noncoding RNA modulate expression. Those factors interact in large, broadly connected regulatory control networks. An engineer following classical principles of control theory would design a simpler regulatory network. Why are genomes overwired? Neutrality or enhanced robustness may lead to the accumulation of additional factors that complicate network architecture. Dynamics progresses like a ratchet. New factors get added. Genomes adapt to the additional complexity. The newly added factors can no longer be removed without significant loss of fitness. Alternatively, highly wired genomes may be more malleable. In large networks, most genomic variants tend to have a relatively small effect on gene expression and trait values. Many small effects lead to a smooth gradient, in which traits may change steadily with respect to underlying regulatory changes. A smooth gradient may provide a continuous path from a starting point up to the highest peak of performance. A potential path of increasing performance promotes adaptability and learning. Genomes gain by the inductive process of natural selection, a trial and error learning algorithm that discovers general solutions for adapting to environmental challenge. Similarly, deeply and densely connected computational networks gain by various inductive trial and error learning procedures, in which the networks learn to reduce the errors in sequential trials. Overwiring alters the geometry of induction by smoothing the gradient along the inductive pathways of improving performance. Those overwiring benefits for induction apply to both natural biological networks and artificial deep learning networks.

## Introduction

What determines gene expression? The list keeps growing: transcription factors, methylation, histone codes, DNA folding, intron sequences, RNA splicing, noncoding RNA, and others ^1, 2^.

Hundreds of genomic variants affect human traits, such as height ^3^. Consider pathways of influence. Numerous factors affect gene expression. Many genes affect a trait. Vast wiring connectivity links genomic influence to a trait.

An engineer following classic principles of control theory would design a simpler system with fewer connections ^4^. Genomes are *overwired*. They have far more nodes and connections than classically engineered systems.

Why are genomes overwired? I discuss possible causes. I then consider wiring density more broadly. What other sorts of systems tend to be overwired?

Computational neural networks in artificial intelligence stand out. Deeply, densely connected computational networks pervade modern life. New computational systems often outperform humans.

The recent computational concepts and methods comprise *deep learning*. The *learning* simply means using data, or past experience, to improve classification of inputs and adjustment of response. The *deep* qualifier refers to the multiple layers of deep and dense network connections ^5, 6^.

That wiring depth, and the computational techniques to use vast connectivity, triggered the revolutionary advances in performance. I discuss genomic wiring in relation to deep learning. I suggest that the inductive systems of biological adaptation and computational learning gain in similar ways from diffusely and densely wired networks.

## Causes

Why do so many factors modulate gene expression? Why is the regulatory network architecture for traits often complex?

### Neutral accumulation

A noncoding RNA may, by chance, alter the expression of various genes. Small modulations of expression may have relatively little effect on fitness. If so, a novel noncoding RNA variant may be effectively neutral. Nearly neutral variants accumulate by chance.

Many nearly neutral variants may accumulate over time. As each variant spreads, it changes the genomic environment of gene regulation. When the aggregate effect of many nearly neutral variants becomes significant, natural selection will retune expression to compensate.

After compensation occurs, one cannot remove the layers of accumulated modulating factors without causing deleterious changes in gene expression. What began as neutral accumulation becomes integral to genomic function. Wiring complexity increases irreversibly.

Lynch’s neutral theory of genome architecture makes predictions ^7, 8^. Smaller population sizes increase chance fluctuations. Greater fluctuations allow larger fitness effects to become nearly neutral. Broader neutrality enhances the rate at which changes accumulate. Smaller populations may tend toward overwiring.

By contrast, large populations more efficiently prune small effects on fitness. Small modulations of gene expression accumulate more slowly. Larger populations may not overwire as readily as smaller populations.

If the fitness effects of modulation tend to be larger, nearly neutral variants will be less common. Prokaryotes may tend to have relatively large deleterious fitness effects of novel modulating factors, because increased genome size and complexity may slow the speed of cellular replication. Eukaryotic genomes may be less sensitive to size and complexity because organismal replication is less strongly coupled to speed of cell division.

Overall, prokaryotes tend to have larger populations and greater sensitivity to genome size and complexity. Such characteristics restrict the scope for neutral accumulation and overwiring. By contrast, eukaryotes tend toward smaller populations and less sensitivity to genome size and complexity. Those characteristics favor neutral accumulation and overwiring. Stronger predictions arise when one can compare closely related organisms that differ in population size and genomic sensitivity.

### Robustness and decay

Modulating factors combine to influence traits. The mechanism of combination matters. Consider two alternatives.

First, suppose modulating factors add together to determine a trait. Then, the more modulating factors, the greater the trait’s variance. Put another way, the more things that cause fluctuations in gene expression, the more variable the trait. In the classical summation model, the variance contribution of each factor is *σ* ^2^. Summing *n* components yields a trait variance of *nσ* ^2^, rising with the number of components.

Second, suppose modulating factors average together to determine a trait ^9^. When averaging *n* components, we divide the effect of each component by *n*. As the number of components rises, the effect of each component declines. Averaging *n* components yields a trait variance of *σ* ^2^ */n*, declining with the number of components.

One can think about each additional modulating component as perturbing trait expression. Robustness is decreased sensitivity to perturbation. In the averaging model, the greater the number of factors, the weaker the effect of each individual perturbing factor. Thus, averaging reduces sensitivity to each perturbation, enhancing robustness.

If modulating factors average together, the benefits of enhanced robustness can favor an increase in the number of factors ^9^. Generally, if the effect of an additional factor causes a sufficient decline in the average contribution of each factor, then natural selection can favor a tendency for the number of factors to increase. Ultimately, many factors of small effect modulate trait expression.

Under the averaging model, evolutionary dynamics follows an interesting path. An additional modulating factor may be favored because it reduces sensitivity to perturbation. Once the new factor is added and sensitivity is reduced, selective intensity against perturbations weakens. Weaker selection allows the accumulation of additional mutations with larger perturbing effects. That shift in mutation-selection balance causes a decay in the average fitness effect of each factor.

Dynamics progresses like a ratchet ^10, 11^. New factors get added for their enhanced robustness. All factors then decay. Taking away a recently added factor exposes the increased deleterious effects of the remaining factors. Exposure of those deleterious effects opposes reversal. One cannot go back.

### Gradient smoothing

Hundreds of genomic variants influence traits, such as human height and weight. Most variants have small effects. Many small effects smooth the gradient of trait values.

A smooth gradient means that a trait may potentially change steadily, or monotonically, with respect to underlying genomic changes. We may think of a smoothly increasing path from a starting point up to the highest peak or down to the lowest valley.

Overwiring leads to many genomic variants of small effect, which in turn smooths the gradient. Thus, we may say that overwiring causes a smooth gradient. What about the converse? Do the benefits of a smooth gradient favor overwiring? Consider three potential benefits.

A smooth gradient enhances adjustability. A densely wired regulatory network has many different connections that can alter traits by a small amount. Such overwired connectivity allows inputs to modulate expression smoothly.

A smooth gradient promotes learning ^12^. Learning requires adjustment in response to input and measurement of success. A system learns as it steadily climbs the gradient of success by smoothly adjusting expression in response to inputs.

A smooth gradient boosts evolutionary adaptability ^13, 14^. Natural selection is essentially a trial and error learning algorithm. The advantages of densely overwired control for learning apply to evolutionary adaptation by natural selection.

The smooth gradient benefits of adjustability, learning, and adaptability can potentially favor overwiring.

## Deep learning

Systems can easily adjust, learn, and evolve if they have smooth gradients. Many of the algorithmic tricks and underlying concepts of machine learning and artificial intelligence come down to how one smooths the gradient ^5, 6^. A smooth gradient provides a steadily improving path from the starting point to an improved target point.

Some biological networks may be densely wired because of the benefits of gradient smoothing. Ideally, we could analyze how network architecture and connectivity strengths affect gradients. However, we do not yet know enough about the details of biological networks. By contrast, the study of computational networks has advanced greatly in recent years. Those advances in computational studies hint at some principles of networks and gradient smoothing. Those principles provide clues about the design of biological networks by natural selection.

Computational networks are loosely modeled after biological neural networks. A set of nodes takes inputs from the environment. Each input node connects to another set of nodes. Each of those intermediate nodes combines its inputs to produce an output that connects to yet another set of nodes, and so on. The final nodes classify the environmental state, possibly taking action based on that classification.

A network learns by altering its parameters ^5, 6^. The parameters set the connection strength between nodes, and how individual nodes combine their many inputs to determine the strength of their output. For example, the input to a network may be an image of a numerical digit. The input nodes are sensors that react to the image. Those sensors initiate activations that pass through all of the connections and layers of the network. The final layer provides a set of ten probabilities, one probability for each of the digits 0, 1, . . . , 9.

The network, when presented with an image of the digit 7, classifies the image by returning a set of ten probabilities. The optimal classification is a probability of one for 7 and zero for all other digits. We can calculate an error distance between the optimal classification and the network’s guess. An error distance is a function of the differences in the probabilities of the optimal and guessed classification.

The error distance can be used to update the network’s parameters. We find a set of small changes in the network parameters that would have yielded a small reduction in the error distance. By following this gradient of improving performance, the network may learn from experience.

That learning approach works as long as there is a smooth path of increasing performance. Improved performance means that the adjustment process truly learns the general features of digit images that enhance future classification. Performance does not improve if adjustments focus on unusual features of the digit images used to train the network. Those unusual features may not be present in many other digit images.

A deep neural network has many layers of nodes between initial inputs and final outputs. Until recently, deep and densely connected computational networks often learned slowly and then got stuck, unable to learn from further information.

Getting stuck often means an unsmooth gradient. Initially, the system learns. It uses past trials to adjust its parameters, yielding a reduction in the error distance for future trials. Then the system gets stuck. Parameter adjustments do not improve future performance.

Put another way, initially the system descended smoothly along the error gradient, improving performance as the error became smaller. Then the gradient flattened out, so that adjustments of the parameters either did not change future error or increased future error.

From that stuck location of parameters, there are no easily discovered altered parameters that follow a smoothly continuing path to a lower point on the error gradient. Other parameter combinations with better performance often exist. But there is no smoothly descending path on the error gradient from the current location to those better combinations.

An improved learning system means a system that smooths the gradient sufficiently, descending on the error gradient to the better locations. The recent revolutionary increase in the performance of deep learning networks arose from a variety of computational adjustments. Many of those adjustments were discovered by trial and error, simply finding that they worked well on real problems ^5, 6^.

For example, limiting the connection strength between nodes prevents dominance by a small set of pathways of connectivity. It seems that broad, densely connected networks that retain many pathways of connectivity have greater learning potential. In essence, a deep, densely and broadly connected network provides a robustly smoothed gradient.

Other adjustments include the functions by which individual nodes combine inputs to determine output. No available theory describes exactly how to construct such functions. Again, trial and error has shown certain functions to work well. Most likely, those successful functions enhance the breadth of pathways that can adjust by small amounts in response to new information, again smoothing the gradient.

Network architecture also affects performance. Architecture includes the number of layers of nodes and the manner in which nodes connect. Connections feed forward from inputs to outputs or feed back from later nodes toward earlier nodes. The feature detectors in the sensory input nodes set the initial representation of environmental states. The network generalizes that low-level representation as information passes through the network layers.

Presumably, architecture and representation ultimately contribute to performance through better gradient smoothing. In a sense, better capacity to learn and better gradient smoothing are nearly the same thing. But the emphasis on gradient smoothing can be useful, because it calls attention to the mechanisms by which particular network properties may contribute to better performance.

Over time, we may come to understand the mechanisms that improve performance and smooth gradients in deep learning networks. We can then consider how those advances in computational networks may provide insight into genomic network architecture, sensory representation, and the consequences for gradient smoothing.

We know that densely connected computational and biological neural networks perform spectacularly at learning, and that densely connected genomic networks perform spectacularly in terms of adjustability and evolvability. We are still trying to understand why (see Appendix for references).

## Geometry of induction

The spectacular performance of large densely wired networks hints at key underlying principles. I conclude by suggesting that large networks are particularly good at smoothing gradients in a way that facilitates induction. Before turning to induction, it is useful to consider deductive principles.

Control theory deduces general principles of wiring to achieve particular design goals ^4^. For example, simple feedback often keeps a system near a setpoint. The setpoint may be a fixed temperature or a fixed concentration. Deviation of the output from the setpoint is fed back to the system as an additional input to the controller. If the feedback signal tells the system that it is below its setpoint, the controller triggers increased output.

Many examples of genomic wiring follow simple feedback ^15– 17^. Other classic control theory motifs also occur frequently in genomic wiring pathways ^18^. The deductive theoretical principles of control successfully predict key aspects of genomic wiring.

However, more complex challenges in engineering and in genomes often seem to be solved by deeply, densely wired networks. I call those networks *overwired*, in the sense that their connectivity patterns are much deeper, denser and broader than predicted by classical deductive principles.

Overwired systems may have embedded within them feedback loops and other classic wiring motifs. But those motifs no longer act alone in a simply interpreted manner. Instead, they are enmeshed within such a large web of diffuse connectivity that it is often difficult to trace their particular effects and functions.

Why do some systems wire simply along classical deductive lines and other systems overwire? I have argued that overwired systems smooth gradients to allow adjustability and adaptability. Put another way, such networks can change in response to experience. A sequence of specific events can lead to improvement of future performance. The networks somehow use their specific experience to find general solutions to a challenge. The networks inductively use specific examples to learn general solutions.

Inductive improvement often requires a smooth gradient. Overwiring may be favored because it enhances the scope for small changes in parameters to descend smoothly along a gradient of decreasing error.

The problem is essentially geometric. How do topological changes in network architecture reshape the error gradient? How do particular bounds on connectivity parameters smooth the gradient? How do particular nodal transformations of inputs into outputs alter gradient shape? How do the input sensors and input representations change the error gradient and consequent inductive performance?

Inductive improvement occurs on various timescales. Over short periods of time, an organism may adjust its response to the environment by changing various parameters within its regulatory network. Over long periods of time, natural selection reshapes the design of the regulatory network. Both short-term adjustments and long-term changes in design arise inductively. Biological systems do not deduce principles. They inductively arrive at abstract representations of environmental challenges. They narrow the error distance along the geometric path of inductive improvement.

Many biological regulatory networks are simple, following closely along classical deductive design principles. In those cases, inductive evolutionary processes discovered those simple deductive principles. Other biological networks are overwired, apparently tuned for inductive potential.

Final questions arise. What sorts of environmental challenges favor classically deductive wiring? What sorts of challenges favor inductive overwiring? What historical aspects of organismal evolution constrain network design? How can we relate deep learning solutions of engineering problems and genomic wiring solutions of biological problems to a more general geometric theory of induction?

## Appendix

Many people have considered the analogies between computational, neural, and evolutionary systems. John Holland at the University of Michigan deeply influenced by own thinking. His 1975 book ^19^, *Adaptation in Natural and Artificial Systems*, provided a focal point at which early artificial intelligence converged with adaptation by natural selection. Holland’s 1986 book ^20^, *Induction: Processes of Inference, Learning, and Discovery*, completed his broad framing of the fundamental problems.

We now understand much more about the particular mechanisms that smooth pathways of inductive progress in learning and evolution. The ideas of artificial intelligence, evolution, and neural computation all have their own vast intellectual histories, with many great contributors. I limit the discussion here to a few key references that illuminate the synergies between disciplines and can provide a start on tracing the intellectual history.

In my own work, I have considered some of the abstract ways in which natural selection may be thought as an inductive process that accumulates information ^21, 22^. Richard Watson and his colleagues have developed ideas more directly relevant to problems of wiring in inductive systems ^23– 25^. Within the field of genomics, various studies have considered how specific evolutionary processes have shaped the wiring patterns of regulatory control pathways. Pastor-Sattorras *et al.* ^26^ provide a good example of that genomic theory.

The smoothness of evolutionary pathways along gradients of biological fitness and the problems of getting stuck have been among the most enduringly controversial issues in theoretical biology ^27^. The topic is often discussed in terms of the Fisher-Wright controversy ^28^. Gavrilet’s book provides a broad discussion of evolutionary gradients ^13^. The shape of the evolutionary gradient has also been related to ways in which gene interactions evolve ^29^, particularly with respect to alternative wirings that are essentially neutral ^30, 31^.
