# Supplementary material for: Puzzles in modern biology. V. Why are genomes overwired?
Source: F1000Res. 2017 Aug 14;6:924. Originally published 2017 Jun 16. [Version 2] doi: 10.12688/f1000research.11911.2 (PMC5580404; doi:10.12688/f1000research.11911.2)
Supplement: Peer review history

# Peer review: Referee response for version 2

**DOI:** 10.5256/f1000research.13383.r24967

**Published:** 2017-08-22

**Authors:** David M. McCandlish

**Affiliations:** 1 Simons Center for Quantitative Biology, Cold Spring Harbor Laboratory, Cold Spring Harbor, NY, USA

I find the author’s revision satisfactory. I understand that the author is writing under several practical constraints, and my assessment is that the new Appendix strikes a good balance between maintaining accessibility in the main text and providing entry points into the literature for the reader who wants to learn more.

 With luck, this series of articles will inspire a new generation of biologists to tackle these important problems.

I have read this submission. I believe that I have an appropriate level of expertise to confirm that it is of an acceptable scientific standard.

# Peer review: Referee response for version 1

**DOI:** 10.5256/f1000research.12874.r23733

**Published:** 2017-07-24

**Authors:** David M. McCandlish

**Affiliations:** 1 Simons Center for Quantitative Biology, Cold Spring Harbor Laboratory, Cold Spring Harbor, NY, USA

The outsides of organisms are most often exquisitely, even ruthlessly, adaptive. Inside the organism’s body, the situation is more heterogeneous. Both physiology and the function of macromolecular complexes are in many instances technically stunning. On the other hand, the wiring diagram of the cell is bedlam. Even based on our current — and likely quite incomplete — state of knowledge, regulatory networks appear to be both more densely and more broadly interconnected than would seem necessary. This is surely a puzzle of modern biology, and Frank has done us a service by cataloging the live hypotheses and pointing us towards the possibility of a resolution wherein this “over-wiring” simply reflects general principles of inductive inference.

 My main criticism of this article is that it does not engage sufficiently strongly with either the contemporary or historical literature. On the contemporary side, it would seem appropriate to directly address the ongoing efforts by several groups to formally link population genetics to general principles of inference (of course, Frank has contributed substantially in this area himself by clarifying the relationship between natural selection and information geometry, see e.g. Frank 2012 “Natural selection V. How to read the fundamental equations of evolutionary change in terms of information theory”). These efforts have been recently reviewed by Watson and Szathmary 2016 in a TREE piece “How can evolution learn?”, which hits on many of the same themes as the latter half current manuscript. Watson’s work in this area seems particularly relevant, and indeed he calls his theory “Evolutionary connectionism” (Watson *et al.* 2015). An important insight from this series of papers is a possible relationship between the evolutionary problem of evolvability and the statistical problem of overfitting. In particular, they suggest that pressure for developmental simplicity can improve the ability of evolutionary systems to generalize in a manner similar to how regularization, drop outs, or early stopping can prevent over-fitting in machine learning (e.g. Kouvaris *et al.* 2017 “How evolution learns to generalize, using the principles of learning theory to understand the evolution of developmental organisation”). The idea that the topology of regulatory networks is a generic consequence of evolution by gene duplication (as in, e.g. the work of Ricard Solé), and more generally by the expansion of gene families, also seems like it deserves a mention as at least a possible proximal cause of over-wiring.

 On the historical side, I think more could be done to link the current discussion with historical themes in evolutionary thought. For instance, the discussion about many possible genomic changes with small effects smoothing the gradient and allowing evolutionary optimization could be put in the context of Fisher and Wright’s disagreements over the structure of fitness landscapes. Wright thought that the reality of building a functional physiology would produce fitness landscapes with many local maxima, so that the key question in evolution was to identify the population-genetic regimes where progress on such a landscape is possible (Wright 1931, 1932). Fisher thought that in high dimensions, these local maxima would largely turn into saddle points, and that in any case, environments were generally changing fast enough that populations were usually chasing a moving optimum rather than adapting on a fixed fitness landscape (Fisher 1930). Frank’s discussion of “getting stuck” in the current manuscript provides additional nuance to this classical disagreement by emphasizing the possibility of extended, high-dimensional plateaus that while strictly speaking are saddle points function in an evolutionary sense more like local optima. The reader interested in resolving this puzzle should also be directed to some of the stone-cold classics in this area such as Wagner and Altenberg 1996 and Stoltzfus 1999 (“On the possibility of constructive neutral evolution”).

I have read this submission. I believe that I have an appropriate level of expertise to confirm that it is of an acceptable scientific standard, however I have significant reservations, as outlined above.

# Response

**Published:** 2017-08-05

**Authors:** Steven Frank

**Affiliations:** Department of Ecology & Evolutionary Biology, University of California, Irvine, USA

I thank David McCandlish for his thoughtful comments and his broad knowledge of the literature. I have added an Appendix that includes the suggested references plus a few others. I agree that there is much prior work and relevant literature. In this series of articles, I am writing to a strict word limit and in a way that can be read by the beginning student as well as the advanced professional. In this case, I perhaps had limited my references too much. The Appendix should provide a start for those who wish to follow up in the literature.

# Peer review: Referee response for version 1

**DOI:** 10.5256/f1000research.12874.r23536

**Published:** 2017-07-03

**Authors:** Sean Nee

**Affiliations:** 1 Department of Ecosystem Science & Management, The Pennsylvania State University, University Park, PA, USA

This is a stimulating, original and thought-provoking and, so, I recommend publication. I see nothing incorrect, so no changes are requested by me as a referee.

 Some thoughts provoked in my mind are as follows.

 First: is engineering really as cut-and-dried as we suppose? A cursory reading of fly-by-wire disasters suggests that the elegant theorems of classical control theory may not be as powerful as one would wish. My impression is that engineers are acutely aware that new technologies such as "cyber physical" systems ^1^, which are most akin to biological systems, are necessitating a complete rethink of the conceptual foundations of their subject matter. Even for more traditional technologies, it is not obvious to me that engineering is as purely “deductive” a subject as we might like to think as we board an aircraft, using the word “deductive” in a way that I may be misconstruing as Frank’s usage.

 Second: to use Dawkin’s convenient metaphor, could a sighted watchmaker really design a “simpler” immune system, for example, than a blind one? If so, is that to do with historical aspects of evolution/population sizes/mutational spectra and so on? If so, what sort of science are we as biologists looking to create: one that says, for example: genome duplication events and large population sizes are responsible for … what? This would be restricting our thinking about evolution to providing explanations of the contrasting failings of different groups of creatures.

 More interesting to me is what I believe Frank is suggesting: the blind watchmaker may have much to teach the sighted ones. This is particularly so in the case of Artificial Intelligences. These are of great interest as both biologists and engineers are only at the starting gate of understanding, and we are all dealing with the question of the design of systems which have a tiny number of component types – “neurons”. I expect to see a unification of psychology and AI engineering in the near future.

 Time will tell whether any notions that may have floated around in classical thinking about evolutionary genetics will advance this program.

I have read this submission. I believe that I have an appropriate level of expertise to confirm that it is of an acceptable scientific standard.

## References

[1] LeeE.A.: Cyber physical systems: Design challenges. *In Object oriented real-time distributed computing (isorc), 2008 11th ieee international symposium on, IEEE.*.2008; 10.1109/ISORC.2008.25 363-369 10.1109/ISORC.2008.25

# Response

**Published:** 2017-08-05

**Authors:** Steven Frank

**Affiliations:** Department of Ecology & Evolutionary Biology, University of California, Irvine, USA

I thank Sean Nee for his thoughtful comments that extend the scope of the discussion. This exchange is included as part of the final publication of my article, so I confine my response to these comments.

Italics quote from Nee’s review.

*First: is engineering really as cut-and-dried as we suppose? ... it is not obvious to me that engineering is as purely “deductive” a subject as we might like to think as we board an aircraft, using the word “deductive” in a way that I may be misconstruing as Frank’s usage.*

I certainly agree that engineering in practice works as much by trial and error as it does by an abstractly pure deduction. I had intended the labeling of engineering design as "deductive" primarily as a comparison with how evolutionary design by natural selection is relatively more "inductive" in character.

Engineers do use deductive principles of control theory to aid in the design of control systems. When novel deductive understanding of control principles arises, engineers readily alter their approach to design, benefitting from the improved general insight. By contrast, natural selection is essentially a purely inductive process. That inductive process cannot throw out a past design and start over, but must improve only by layering small inductive gain upon small inductive gain.  

*Second: to use Dawkin’s convenient metaphor, could a sighted watchmaker really design a “simpler” immune system, for example, than a blind one? ... More interesting to me is what I believe Frank is suggesting: the blind watchmaker may have much to teach the sighted ones. This is particularly so in the case of Artificial Intelligences.*

I meant the comparison in both of the ways that Nee discusses. A sighted watchmaker would make a different immune system from a blind watchmaker. Whether that different immune system of the sighted watchmaker would be simpler or better is hard to say. However, I suspect that it would be simpler because humans tend to design systems that they can analyze and understand, whereas blind induction does not care about the logic or the complexity of the mechanism.

I agree with the latter aspect in Nee's comments: that the blind watchmaker provides new insights about design that the sighted watchmaker may consider. We are seeing this now in the great advances in artificial intelligence: the goal of the sighted watchmaker has become to improve the ways in which the blind watchmaker's trial and error induction proceeds.
